# Medical Management of Cerebral Vasospasm following Aneurysmal Subarachnoid Hemorrhage: A Review of Current and Emerging Therapeutic Interventions

**DOI:** 10.1155/2013/462491

**Published:** 2013-04-15

**Authors:** Peter Adamczyk, Shuhan He, Arun Paul Amar, William J. Mack

**Affiliations:** Department of Neurological Surgery, Keck School of Medicine, University of Southern California, Los Angeles, CA 90033, USA

## Abstract

Cerebral vasospasm is a major source of morbidity and mortality in patients with aneurysmal subarachnoid hemorrhage (aSAH). Evidence suggests a multifactorial etiology and this concept remains supported by the assortment of therapeutic modalities under investigation. The authors provide an updated review of the literature for previous and recent clinical trials evaluating medical treatments in patients with cerebral vasospasm secondary to aSAH. Currently, the strongest evidence supports use of prophylactic oral nimodipine and initiation of triple-H therapy for patients in cerebral vasospasm. Other agents presented in this report include magnesium, statins, endothelin receptor antagonists, nitric oxide promoters, free radical scavengers, thromboxane inhibitors, thrombolysis, anti-inflammatory agents and neuroprotectants. Although promising data is beginning to emerge for several treatments, few prospective randomized clinical trials are presently available. Additionally, future investigational efforts will need to resolve discrepant definitions and outcome measures for cerebral vasospasm in order to permit adequate study comparisons. Until then, definitive recommendations cannot be made regarding the safety and efficacy for each of these therapeutic strategies and medical management practices will continue to be implemented in a wide-ranging manner.

## 1. Introduction

Aneurysmal subarachnoid hemorrhage (aSAH) occurs in approximately 30,000 patients in the United States each year [1]. Cerebral vasospasm is estimated to occur in up to 70% of all aSAH patients and remains a major cause of morbidity and mortality [2]. The complex cascade of factors and events that result in arterial narrowing has been subject to extensive research, leading to a vast array of proposed treatment methods. A large number of these experimental therapies have been evaluated at the basic and translational levels with fewer reported prospective randomized clinical trials. Despite these efforts, no single treatment modality has proven efficacious and trial results have been frequently mixed or conflicting. Therefore medical management practices are often wide-ranging with an assortment of strategies implemented in various permutations. In this report, we review the literature and provide a concise, updated summary of recent clinical trials and current medical treatments evaluated in patients with cerebral vasospasm secondary to aSAH.

## 2. Triple-H Therapy

The current mainstay for medical management of vasospasm secondary to aSAH remains triple-H therapy. The protocol is defined by hypertension, hypervolemia, and hemodilution, often with added hyperdynamic treatment [3]. This strategy is intended to augment cerebral blood flow via expansion of intravascular volume and reduction of blood viscosity. Hypertension may be achieved by volume expansion alone or with the addition of vasopressor medications such as phenylephrine or dopamine. Enhancing volume status may increase cardiac output, resulting in increased vascular resistance and maintenance of cerebral blood flow in hypoperfused territories. Hemodilution remains the least clearly defined component of triple-H therapy. A hematocrit goal of 30–35% has been suggested as an optimal balance between oxygen-carrying capacity and blood viscosity [4, 5]. Caution is required when initiating triple-H therapy as potential complications include cardiopulmonary failure, exacerbation of cerebral edema, renal failure, hyponatremia, sepsis, and a theoretical risk of untreated aneurysm rupture [6, 7].

Triple-H therapy has gained widespread acceptance despite a paucity of large-scale, prospective clinical trials. Moreover, significant variances in administration methods hinder direct comparisons among study results. In a small, randomized trial of aSAH patients waiting to undergo surgical clip ligation, those who were managed with centrally acting antihypertensive medications or vasodilators demonstrated a significant reduction in vasospasm (*P* < 0.01) and increase in preoperative survival rate (87% versus 53%, *P* < 0.01) when compared to those managed with diuretics and volume restriction [8]. Although fluid restriction appears to be associated with less favorable outcomes, there has been little evidence suggesting superiority of hypervolemia when compared to euvolemia. Lennihan et al. evaluated 82 aSAH patients who were randomized to receive either hypervolemia or euvolemia following surgical clipping (until postbleed day 14). While hypervolemic therapy increased cardiac filling pressures and fluid intake, neither cerebral blood flow nor cerebral blood volume parameters improved. The incidence of cerebral vasospasm was 20% in each group. Further, no significant differences were observed in clinical outcomes at one year [9]. Another small prospective, randomized clinical trial that enrolled 32 patients reported no significant differences in the rate of cerebral vasospasm or clinical outcomes at one year in patients randomized to triple-H versus euvolemic therapy. Moreover, patients treated with triple-H therapy experienced more complications and incurred higher medical costs [5]. By contrast, a 2003 meta-analysis reviewed four prospective trials of triple-H therapy in 488 patients and reported significant reductions in symptomatic vasospasm incidence (RR = 0.45, CI = 0.32–0.65) and mortality (RR = 0.68, 95% CI = 0.53–0.87) for patients who received triple-H therapy. However, treatment was not associated with a reduction in delayed ischemic neurological deficits. The authors concluded that there remains insufficient data to make recommendations regarding utilization of prophylactic triple-H therapy [3]. A recent systematic review of the different triple-H therapy components suggested that induction of hypertension is more effective in increasing cerebral blood flow than hemodilution or hypervolemia alone [10].

Based on the previous findings, recent American Heart Association guidelines suggested maintenance of euvolemia for vasospasm prevention and recommended induced hypertension for patients with active cerebral vasospasm. Furthermore, recommendations advised against induction of hypervolemia prior to radiographic evidence of vasospasm [11].

Lack of evidence-based standards with regard to hemodynamic endpoints or utilization of specific therapeutic agents has generated substantial practice variation. A recent survey of neurocritical care providers revealed that 27% still induced prophylactic hypervolemia. This practice was more likely instituted in centers lacking a dedicated neurointensive care unit. Only 12% reported prophylactic induction of hypertension, although all respondents recommended therapeutic hypertension in patients with severe TCD or angiographic vasospasm associated with clinical symptoms. The reported goal SBP ranged from 140 to 240 mmHg, with an associated MAP ranging from 70–210 mmHg [12]. In the same survey, up to 41% of respondents reported incorporation of hyperdynamic therapies, primarily with dobutamine and milrinone, to increase cardiac output in the presence of severe cerebral vasospasm.

Randomized trials examining the utility or parameters of dobutamine treatment are lacking. One small study demonstrates measured increases in cerebral blood flow [13] and another reports reversal of ischemic deficits in patients unresponsive to fluid therapy [14]. Milrinone is another hyperdynamic agent that acts through selective inhibition of low-Km cAMP specific phosphodiesterase III. It exhibits both inotropic and vasodilatory effects. A combination of intra-arterial and intravenous milrinone has been investigated in several small studies focusing, primarily, on endovascular outcomes. Despite improvement in angiographic vasospasm, a substantial number of patients experienced systemic hypotension and vasospasm recurrence [15, 16]. The variability and shifting emphasis of triple-H therapy highlight the potential benefit for further sufficiently powered randomized controlled trials to adequately investigate this treatment modality.

## 3. Calcium Channel Blockers

Although multiple calcium channel blockers have been investigated as therapies for cerebral vasospasm, randomized trials have primarily focused on treatment with nimodipine and nicardipine. The most recent meta-analysis evaluated 16 studies, including 3361 patients. The authors found that calcium channel blockers reduced the risk of poor outcome (RR = 0.81, 95% CI = 0.72–0.92), with benefits largely dependent on oral nimodipine administration [17].

Nimodipine is a dihydropyridine calcium antagonist that blocks calcium influx through L-type calcium channels and possesses regional selectivity for vascular smooth muscle. Oral nimodipine administration has become standard therapy in the setting of aSAH after several randomized trials have demonstrated improved outcomes. Nimodipine currently remains the only drug approved by the US Food and Drug Administration for cerebral vasospasm treatment [18–28]. It does not significantly reverse angiographic vasospasm. Alternate putative mechanisms include vascular effects, such as decreased small vessel resistance with pial collateral augmentation, as well as neuroprotection via reduction of calcium-mediated excitotoxicity [29].

Nimodipine has been administered orally in the majority of clinical trials. The largest randomized study, the British Aneurysm Nimodipine Trial, demonstrated a 34% reduction in cerebral infarction (95% CI 13–50) and a 40% reduction in poor outcome (95% CI 20–55) at three months in the oral Nimodipine group when compared to the placebo cohort [19]. This finding parallels results from the most recent meta-analysis that evaluated 8 prospective randomized trials including 1514 patients. The investigators demonstrated that, in comparison to placebo, nimodipine significantly reduced the incidence of delayed neurological deficits by 38% (OR 0.62, 95% CI 0.50–0.78) and cerebral infarcts by 48% (OR 0.52, 95% CI 0.41–0.66) [30]. Additionally, a retrospective analysis found nimodipine to be a safe and cost-effective treatment that increases life expectancy with low incremental costs [20].

Nicardipine is an alternate dihydropyridine calcium antagonist that is delivered parenterally and exhibits regional selectivity on vascular smooth muscle. Initial studies suggested that nicardipine administration resulted in both angiographic vasospasm reduction and symptomatic improvement [31]. However, a subsequent prospective randomized trial, the Cooperative Aneurysm Study, found that while nicardipine treatment significantly reduced vasospasm incidence (32% versus 46%, *P* = 0.001), 3-month clinical outcomes (Glasgow Outcome Scale (GOS)) were comparable to those of the placebo group [32]. The efficacy of nicardipine has been evaluated via alternative delivery methods. A small prospective randomized study found that placing nicardipine prolonged-release implants into the basal cisterns during surgical clipping results in reduced angiographic vasospasm (*P* < 0.05) and improved modified Rankin Scale scores at 1 year (*P* = 0.0001) [33]. A recent, small, retrospective case-control study evaluated 14 patients who underwent intraventricular delivery of nicardipine. The study suggested evidence of safety and TCD flow velocity reduction, but no significant change in 3 month clinical outcome [34].

Fasudil is a more recently developed calcium channel blocker, primarily studied in Japan, that prevents endothelial narrowing via inhibition of the Rho-kinase signaling pathway [35]. One randomized clinical trial exhibited significant reductions in angiographic vasospasm (*P* = 0.002), symptomatic vasospasm (*P* = 0.025), and radiographic infarcts (*P* = 0.001) with improved one-month clinical outcomes (*P* = 0.015) among the 131 patients who were randomized to receive IV fasudil for 14 days when compared to those who received placebo [36]. Another recent trial randomized 63 patients to IV fasudil and 66 patients to IV nimodipine treatment. The incidence of symptomatic vasospasm and radiographic infarcts remained similar in both groups. However, a higher proportion of patients who received IV fasudil demonstrated good one-month clinical outcomes (74.5% versus 61.7%, *P* = 0.040) [37]. A 2012 meta-analysis evaluating 8 studies found that IV fasudil administration resulted in a significant reduction in angiographic vasospasm (OR = 0.40, CI 0.24–0.67, *P* = 0.0004) and vasospasm-related infarcts (OR = 0.43, CI 0.26–0.70, *P* = 0.0008) when compared to placebo treatment. The authors concluded that fasudil remains a promising therapeutic agent that requires further validation with large randomized controlled trials [38].

## 4. Magnesium

The efficacy of calcium channel blockers in the setting of aSAH has led to studies of magnesium, a divalent cation that binds voltage-dependent calcium channels to inhibit smooth muscle contraction. Magnesium may also exhibit neuroprotective benefits by inhibiting glutamate release. In one small randomized controlled trial, 20 patients who received IV magnesium demonstrated a trend (nonsignificant) towards reduction in mean transcranial Doppler velocities and improvement in Glasgow outcome scores when compared to those who received placebo treatment [39]. Another small trial noted a nonsignificant reduction of symptomatic vasospasm (43% versus 23%, *P* = 0.06), in addition to significant reductions in the duration of TCD elevations (*P* < 0.01) in 30 patients who were randomized to IV magnesium infusion compared to those randomized to placebo therapy [40]. Muroi et al. found a nonsignificant trend towards improved 12-month clinical outcomes (*P* = 0.083) for patients who received IV magnesium infusion but noted significant side effects including hypocalcemia (*P* = 0.005) and hypotension (*P* = 0.04) [41]. The magnesium in Aneurysmal Subarachnoid Hemorrhage (MASH) trial that was a larger randomized trial demonstrated nonsignificant reductions in delayed cerebral ischemia (34%, HR = 0.66, CI = 0.38–1.14) as well as 3-month poor clinical outcomes (23%, RR = 0.77, CI = 0.54–1.09) among 283 patients who received a 14-day continuous IV magnesium infusion [42]. 

Trends towards improved outcomes in these trials led to more recent, larger investigations that ultimately failed to demonstrate substantial benefit from IV magnesium therapy. The intravenous magnesium sulphate for Aneurysmal Subarachnoid Hemorrhage (IMASH) trial demonstrated nearly identical 6-month outcomes in 169 patients who were randomized to continuous IV magnesium therapy and those randomized to placebo treatment [43]. The largest and most recent phase III randomized trial, the MASH-2 trial, revealed similar outcomes. Additionally the investigators conducted a meta-analysis of seven randomized trials involving 2047 patients. They demonstrated no reduction in the incidence of poor outcomes for patients treated with magnesium therapy (RR 0.96, CI 0.86–1.08). These findings prompted the authors to recommend against the use of IV magnesium for cerebral vasospasm in patients with aSAH [44]. 

## 5. Statins

Hydroxymethylglutaryl coenzyme A reductase inhibitors, commonly referred to as “statins,” may reduce cerebral vasospasm through multiple putative mechanisms, including endothelial protection and anti-inflammatory effects.

In a randomized controlled trial, patients who received oral pravastatin demonstrated a 32% reduction in TCD-diagnosed vasospasm when compared to those receiving placebo treatment (*P* = 0.006). Further, the pravastatin cohort exhibited decreases in vasospasm-related ischemic deficits (*P* < 0.001) and mortality (*P* = 0.037) [45]. In a smaller randomized trial, vasospasm was also reduced in patients treated with simvastatin when compared to those administered placebo (26.3% versus 60%, *P* < 0.05) [46]. A meta-analysis of randomized statin trials, including 158 patients, noted statistically significant reductions in vasospasm (RR = 0.73, CI = 0.54–0.99), ischemic neurological deficits (RR = 0.38, CI = 0.17–0.83), and mortality (RR = 0.22, CI = 0.06–0.82). However, methods of vasospasm diagnosis varied substantially among included studies, potentially limiting interpretation of collective results [47].

More recent randomized trials have revealed less promising results. In one trial of 39 patients randomized to simvastatin or placebo, nonsignificant reductions in angiographically confirmed vasospasm and cerebral ischemia were observed for simvastatin-treated patients [48]. Another randomized simvastatin trial demonstrated no difference in TCD-defined vasospasm or clinical outcome at 6-month followup [49]. The most recent meta-analysis, reported in 2010, included 309 patients. The investigators found that statins significantly reduced the occurrence of ischemic neurological deficits (OR 0.38 (0.23–0.65); *P* < 0.001), but not mortality (OR 0.51 (0.25–1.02); *P* = 0.06) or poor neurological recovery (OR 0.81 (0.49–1.32); *P* = 0.39) [50].

The conflicting data underscores a need for larger trials to determine the efficacy of statins in the setting of aSAH. A number of phases II and III trials remain underway, including the Simvastatin in Aneurysmal Subarachnoid Hemorrhage (STASH) trial (NCT00731627), Statins and Cerebral Blood Flow in SAH trial (NCT00795288), and High-dose Simvastatin for Aneurysmal SAH trial (NCT01077206).

## 6. Endothelin Receptor Antagonists

Endothelin receptor antagonists have been designed to act on vascular smooth muscle cells and inhibit the binding of endothelin 1, a potent vasoconstrictive peptide. Both Clazosentan (AXV-034343) and TAK-044 have been studied in patients with cerebral vasospasm. In the largest controlled trial, 402 patients were randomized to receive either IV TAK-044 or placebo. A trend toward decreased ischemic deficits (29.5% versus 36.6%, RR = 0.8, CI = 0.61–1.06) was evident in patients who received TAK-044, although no differences in the 3-month Glasgow outcome scores were demonstrated [51].

Vajkoczy et al. conducted a randomized trial of Clazosentan in the setting of aSAH. The study revealed a decrease in angiographic vasospasm in patients treated with Clazosentan when compared to placebo therapy (40% versus 88%, *P* = 0.008). Results also suggested a nonsignificant trend toward infarct reduction [52]. The Clazosentan to Overcome Neurological Ischemia and Infarction Occurring After Subarachnoid Hemorrhage (CONSCIOUS-1) trial was a randomized dose-escalation study that demonstrated significant reduction in moderate and severe angiographic vasospasm in the Clazosentan cohort (RR = 0.65, CI = 0.47–0.78, *P* < 0.0001) at day 9. Post hoc analysis demonstrated nonsignificant reductions in infarctions for patients who received clazosentan. However, these changes were accompanied by an increase in pulmonary complications, hypotension, and anemia [53]. A follow-up trial, CONSCIOUS 2, found that, when compared to placebo, treatment with Clazosentan had no significant effect on mortality, vasospasm-related morbidity, or functional outcome in patients who underwent surgical clipping. Moreover, patients receiving Clazosentan demonstrated increased pulmonary complications, anemia, and hypotension [54]. CONSCIOUS-3 was a randomized trial evaluating Clazosentan in subarachnoid hemorrhage patients who underwent endovascular coil embolization. This trial was halted prematurely following the completion of CONSCIOUS-2. Results indicated a reduction in vasospasm-related morbidity/all-cause mortality in patients receiving a higher dose of Clazosentan (OR, 0.474; 95% CI, 0.275–0.818; *P* = 0.007). However, improvements in clinical outcomes were not evident [55].

A recent systematic review examined 2601 patients treated with endothelin receptor antagonists. The authors noted that these therapies tended to increase risk of poor functional outcome (RR, 1.12; CI, 0.97–1.28) despite decreased incidence of angiographic vasospasm (RR, 0.58; CI, 0.48–0.71). Endothelin receptor antagonists increased the risk of pulmonary edema, hypotension, and anemia, leading the authors to argue against their use in patients with subarachnoid hemorrhage [56].

## 7. Nitric Oxide

Nitric oxide depletion has been associated with reduction in vasodilatory response. This relationship has prompted interest in vasospasm treatments that increase nitric oxide synthesis. Thomas et al. investigated prophylactic intrathecal delivery of sodium nitroprusside, a direct nitric oxide donor, in a small cohort of patients with aneurysmal SAH. Angiographic improvement was noted in five of six patients with vasospasm. No patients suffered neurological deficits [57]. A more recent report documented TCD velocity improvements in patients with severe cerebral vasospasm treated with intraventricular sodium nitroprusside [58]. By contrast, Raabe et al. found high variability in both treatment efficacy and duration when intraventricular sodium nitroprusside was administered to patients with severe refractory cerebral vasospasm. The authors suggested that increased efficacy may depend on earlier initiation, prior to the onset of vasospasm and continuous administration of sodium nitroprusside [59].

Upon contact with deoxyhemoglobin, nitrites possess potential vasodilatory capacity via nitric oxide production. Pluta et al. demonstrated that a 14-day continuous intravenous infusion of sodium nitrite prevented vasospasm a primate SAH model [60]. The authors recently conducted a safety and feasibility study demonstrating that sodium nitrite could be safely administered in adult patients at defined concentrations [61]. Results from a phase II trial of nitrite therapy following SAH (Safety and Pharmacokinetic Evaluation of Nitrite for Prevention of Cerebral Vasospasm (NCT00873015)) are pending.

Sildenafil is a phosphodiesterase inhibitor that promotes vasodilatation by acting on the nitric-oxide-cyclic GMP pathway. A recent feasibility study assessed sildenafil administration in 72 patients with refractory symptomatic vasospasm. The authors demonstrated temporary TCD improvements in 4 patients and sustained symptomatic relief in 8 patients. Four patients developed complications significant enough to warrant termination of therapy. Encouraging results, however, have generated continued interest in this potential treatment. The Sildenafil for Prevention of Cerebral Vasospasm (SIPCEVA) study is an ongoing phase II trial. (NCT 01091870) [62].

## 8. Free Radical Scavengers

Deleterious free radical byproducts, generated from oxyhemoglobin metabolism, have been implicated in the pathogenesis of cerebral vasospasm. Detailed mechanisms have not yet been fully established. Tirilazad is a 21-aminosteroid that inhibits lipid peroxidation and scavenges free radicals. A large, international, randomized trial enrolled 1015 patients and demonstrated reduced mortality (*P* = 0.01) and improved 3-month clinical outcomes (*P* = 0.01) in patients receiving tirilazad when compared to those receiving placebo treatment. Further, the authors observed a nonsignificant reduction in symptomatic vasospasm, with increased benefit in males compared to females [63]. Conversely, a similar North American trial randomized 897 patients to receive low-dose tirilazad, high-dose tirilazad, or placebo treatment and found no difference in symptomatic vasospasm incidence or 3-month outcomes [64].

Lanzino et al. hypothesized that, due to gender-related pharmacokinetics, benefits might be more apparent in women with administration of larger doses. This led to an international multicenter trial enrolling 819 women randomized to high-dose tirilazad or placebo treatment. Patients who received tirilazad demonstrated reduced symptomatic vasospasm (33.7% versus 24.8%, *P* = 0.005) and decreased infarcts (8% versus 13%, *P* < 0.04) when compared to those receiving placebo. No significant difference in 3-month mortality rate was noted [65]. A similar North American study enrolled 823 women and found no significant differences in symptomatic vasospasm or clinical outcome between patients who received tirilazad and those who received placebo. Benefit was only noted in a subset of patients presenting with WFNS grades IV and V SAH who demonstrated reduced mortality rates relative to placebo-treated patients (*P* = 0.016) [66]. A recent meta-analysis, that included 3821 patients, found that individuals who received tirilazad demonstrated reduction in symptomatic vasospasm (OR 0.80, CI 0.69 to 0.93) but no reduction in unfavorable clinical outcomes or mortality [67].

Nicaraven is a hydroxyl radical scavenger that was evaluated in a prospective randomized trial of 162 patients. In comparison to patients receiving placebo, those randomized to receive intravenous nicaraven demonstrated significant reductions in the incidence of delayed ischemic neurological deficits (*P* < 0.05), poor one-month outcomes (*P* < 0.05), and overall mortality (*P* < 0.05) [68]. Ebselen is a seleno-organic compound with antioxidant activity via a glutathione peroxidase-like mechanism. Saito et al. failed to observe a significant reduction in vasospasm-related neurological deficits in 52 patients who were randomized to receive oral ebselen compared to those who received placebo treatment. However, clinical outcomes were significantly improved (*P* = 0.005) and a corresponding decrease in both the incidence and extent of radiographic infarcts (*P* = 0.032) was noted [69]. More recently, a new free radical scavenger, edaravone, was assessed in a randomized study. Results suggested a nonsignificant reduction in vasospasm-related deficits. Further, the authors noted a significant reduction in the incidences of vasospasm-induced cerebral infarction (0% versus 66%, *P* = 0.028) and poor 3-month outcome scores (0% versus 71%, *P* = 0.046) in the edaravone cohort when compared to those in the placebo group [70].

## 9. Thromboxane Inhibitors

The inhibition of platelet-derived thromboxane A2, which possesses regional vasoconstrictive and prothrombotic effects, has been investigated as a therapy for cerebral vasospasm. A 1989 study observed significant reductions in vasospasm incidence and radiographic infarcts among patients randomized to receive ozagrel (OKY-046), a selective thromboxane synthetase inhibitor [71]. Additional studies have been limited, principally, to Japanese centers. Some Japanese facilities have incorporated the treatment as standard therapy. The most recent publication, in 2008, compared 1138 patients who received ozagrel in combination with fasudil to 3690 patients treated with fasudil alone. Clinical outcomes were found to be similar. Interestingly, patients who received fasudil alone demonstrated a decreased incidence of radiographic infarcts (27.3% versus 32.4%, *P* < 0.01) and reduced symptomatic vasospasm rates (25.7% versus 32.0%, *P* < 0.01) compared to those on combination therapy. Interpretation of the results requires caution, as this investigation was a postmarketing surveillance study for fasudil and was not randomized [72].

## 10. Anti-Inflammatory Treatments

During the acute phase of subarachnoid hemorrhage, serine proteases activate the complement pathway. Serial cleavage initiates an inflammatory cascade implicated in the pathogenesis of cerebral vasospasm. Nafamostat mesilate (FUT-175) is a serine protease inhibitor that suppresses thrombin and plasmin and decreases inflammation. One retrospective case control study evaluating 23 patients who received IV FUT-175 demonstrated a decreased incidence of delayed cerebral ischemia (13% versus 55%, *P* < 0.05) and lower number of cerebral infarctions (9% versus 43%, *P* < 0.05) compared to controls [73]. Kaminogo et al. administered a combination of nafamostat and ozagrel to 34 patients with severe SAH. They demonstrated a lower incidence of symptomatic vasospasm when compared to those given ozagrel alone (*P* < 0.02). A nonsignificant improvement in outcome was also noted. Values reached statistical significance when Hunt and Hess grade 5 SAH patients were excluded. The authors concluded that an additive therapeutic effect can be achieved when nafamostat is administered with ozagrel in the setting of aSAH [74].

Implication of cell-mediated inflammation in the pathogenesis of cerebral vasospasm has led to an interest in steroids as protective agents. Manno et al. administered intravenous cyclosporine in nine patients with severe subarachnoid hemorrhage and found that it failed to prevent vasospasm. Of note, no significant systemic complications were noted [75]. A small case-control study evaluated ten patients treated with topical dexamethasone after undergoing surgical clipping. Reduced vasospasm and lower mean middle cerebral artery TCD velocities were noted in the treated group [76]. One retrospective study assessed high-dose methylprednisolone administration in 21 patients. Results suggested a nonsignificant decrease in symptomatic vasospasm and a significant increase in excellent outcomes (71% versus 29%, *P* < 0.01) in the steroid cohort [77]. In 2010, Gomis et al. randomized 49 patients to intravenous methylprednisolone therapy or placebo treatment and reported a decreased incidence of poor 12-month outcomes (15% versus 34%, CI 0.5–37.9%) in the methylprednisolone group. Outcome improvement was evident despite a lack of significant differences in symptomatic vasospasm [78].

## 11. Thrombolysis

Subarachnoid blood volume is an independent predictor of cerebral vasospasm. This finding has prompted investigations focused on the potential of thrombolytic agents to augment blood clearance from the cerebrospinal fluid following aSAH. One randomized controlled trial found a 56% relative risk reduction of severe vasospasm in 51 patients that received a single intracisternal bolus of tPA after surgical clipping (*P* = 0.02) when compared to those who received placebo injection [79]. Another randomized trial evaluated a single dose of intrathecal urokinase into the cisterna magna in patients who underwent aneurysm coiling. Patients randomized to intrathecal urokinase exhibited a significant reduction in symptomatic vasospasm (8.8% versus 30.2%, *P* = 0.012) as well as significantly improved 6-month outcomes (*P* = 0.036) when compared to the placebo-treated cohort [80].

A 2004 meta-analysis including 652 patients enrolled in nine trials found absolute risk reductions of 14.4% (*P* < 0.001) for delayed ischemic neurological deficits, 9.5% (*P* < 0.01) for poor outcomes, and 4.5% (*P* < 0.05) for mortality among patients treated with thrombolytics. The overall rate of complications secondary to thrombolysis was considered low. No therapeutic difference was found between urokinase and tPA. A significant proportion of studies included in the meta-analysis were not randomized. Therefore, caution should be exercised in interpreting the results. The findings will likely prompt interest in larger randomized trials [81].

## 12. Neuroprotection and Other Treatments

Agents found to demonstrate neuroprotective effects in the context of ischemic stroke and traumatic brain injury have been evaluated in subarachnoid hemorrhage trials. Dantrolene, a ryanodine receptor calcium channel antagonist, inhibits intracellular calcium release and exhibits neuroprotective effects. A pilot investigation evaluated ten patients with cerebral vasospasm who either received high- or low-dose intravenous dantrolene. The study found a significant reduction in peak systolic TCD velocities (−25 cm/s, CI −47 to −5 cm/s, *P* = 0.02) in the low-dose group. Decreased systolic blood pressures were a noted side effect [82, 83].

Erythropoietin (EPO) has exhibited neuroprotective effects in experimental animal models via antiapoptotic, antioxidative, angiogenic, and neurotrophic mechanisms. A 2007 randomized trial evaluated 73 SAH patients treated with intravenous EPO. Analysis demonstrated no significant differences in symptomatic vasospasm incidence or six-month functional outcomes [84]. More recently, a trial conducted in 2009 examined 80 SAH patients randomized to EPO therapy or placebo treatment. Results revealed no differences in overall incidence of vasospasm but suggested a greater number of favorable discharge outcomes (*P* = 0.039) and decreased incidences of severe vasospasm (7.5% versus 27.5%, *P* = 0.037) and cerebral infarcts (7.5% versus 40.0%, *P* < 0.001) in the treatment group. EPO administration appeared well tolerated, prompting further dose escalation trials.

Enoxaparin is an unfractionated heparin derivative. Its selective inhibition of smooth muscle cell migration and proliferation, as well as reduction in free radical production, has been associated with cerebral blood flow augmentation in animal models. One single center study in 2003 randomized 170 patients to receive subcutaneous enoxaparin or placebo. The investigators found no significant difference in three-month outcomes. Moreover, four patients (4.7%) receiving enoxaparin had additional intracranial hemorrhages [85]. A later prospective randomized study of 120 patients utilized a lower dose of subcutaneous enoxaparin. The treatment group exhibited significantly fewer ischemic deficits (8.8% versus 66.7%, *P* < 0.001) and vasospasm-related infarcts (3.5% versus 28.3%, *P* < 0.001) compared to patients who received placebo. Additionally, patients in the enoxaparin group demonstrated fewer intracranial hemorrhages with better 12-month outcomes [86]. In addition to its potential neuroprotective effects, subcutaneous enoxaparin confers benefit in preventing deep venous thrombosis. Taken together, these apparent benefits may encourage further investigation in patients with subarachnoid hemorrhage.

Human albumin has been shown to exert neuroprotective effects in animal models of cerebral ischemia and clinical studies examining a range of intracranial pathologies. The Albumin in Subarachnoid Hemorrhage (ALISAH) trial is a pilot study that evaluated 47 adults treated with escalating doses of 25% human albumin. The investigators noted a trend towards reduction in cerebral infarctions and improved 3-month outcomes with increasing doses. An absence of major complications was reported. Results led to the ALISAH II study, a Phase III, randomized, placebo-controlled trial that remains underway [87].

## 13. Conclusion

Cerebral vasospasm after aSAH is likely multifactorial in etiology. This hypothesis is corroborated by the vast array of available treatment modalities. Currently, the strongest evidence supports use of prophylactic oral nimodipine and initiation of triple-H therapy for patients in cerebral vasospasm. Although these treatments have improved prognosis for aSAH patients, neurological outcomes remain poor in the setting of delayed cerebral vasospasm. This review highlights emerging medical strategies. Large randomized controlled trials are required before definitive recommendations can be made regarding safety and efficacy for each of these treatments. Research efforts may be limited by the uncertain definition of cerebral vasospasm and the differing modalities used to assess this entity. Variability in outcome measurements hinders comparison of trial data. Additionally, studies that incorporate the currently accepted clinical measures require very high sample sizes to demonstrate efficacy and are therefore cost and time consuming [88]. Efforts to make outcome tools more consistent for upcoming trials are underway [89]. Further, limitations have prompted explorations of surrogate biomarkers.

Another important future research consideration is the discrepancy between radiographic vasospasm and delayed neurological deficits. Evidence suggests that treatment of radiographic vasospasm is not always sufficient to improve clinical outcome. These findings support the emerging concept of *delayed cerebral ischemia* that incorporates purported effects beyond those of large vessel narrowing. Microvascular dysfunction and complex neuronal-glial interactions likely both affect cerebral ischemia in the setting of aSAH [90]. Future therapeutic strategies certainly necessitate a more complete understanding of the mechanisms that contribute to overall neurological injury in aSAH.
